# Stress relaxation study of fillers for directly compressed tablets

**DOI:** 10.1016/j.powtec.2011.11.011

**Published:** 2012-02

**Authors:** M. Rehula, R. Adamek, V. Spacek

**Affiliations:** a Department of Pharmaceutical Technology, Faculty of Pharmacy in Hradec Králové, Charles University in Prague, Czech Republic; b Synpo, Pardubice, Czech Republic

**Keywords:** Stress relaxation, Viscoelasticity, van der Waals bonds, Hydrogen bonds, Fillers

## Abstract

It is possible to assess viscoelastic properties of materials by means of the stress relaxation test. This method records the decrease in pressing power in a tablet at its constant height. The cited method was used to evaluate the time-dependent deformation for six various materials: microcrystalline cellulose, cellulose powder, hydroxypropyl methylcellulose, mannitol, lactose monohydrate, and hydrogen phosphate monohydrate. The decrease in pressing powering of a tablet during a 180 s period was described mathematically by the parameters of three exponential equations, where the whole course of the stress relaxation is divided into three individual processes (instant elastic deformation, retarded elastic deformation and permanent plastic deformation). Three values of the moduli of plasticity and elasticity were calculated for each compound. The values of elastic parameters A_Ti_ have a strong relationship with bulk density. The plastic parameters P_Ti_ represent particle tendency to form bonds. The values of plasticity in the third process P_T3_ ranged from 400 to 600 MPas. Mannitol had higher plasticity and lactose monohydrate on the contrary reduced plasticity. A linear relation exists between A_T3_ and P_T3_ for the third process. No similar interpretation of moduli calculated on the basis of three exponential equations has been realized yet.

## Graphical abstract

It was found linear dependence of the parameter P_T3_ and A_T3_. P_T3_ = − 49.91849 + 69.78869A_T3_.

**Figure:**
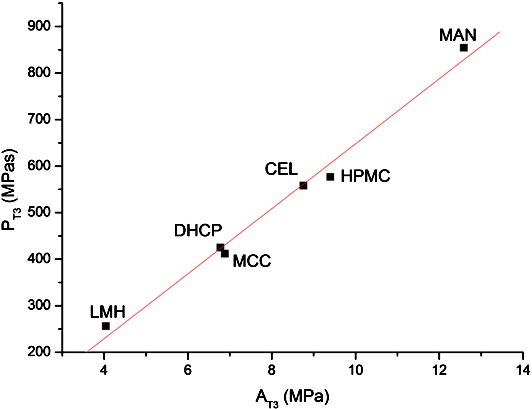

**Highlights:**

► Stress relaxation test was used for the description of viscoelasticity of fillers. ► CaHPO_4_·2H_2_O and microcrystalline cellulose had similar moduli of plasticity. ► A linear dependence of the parameter P_T3_ and A_T3_ was found.

## 1 Introduction

Tablets were prepared by a pressing operation of excipients plastically deformable. The excipients' plasticity is evaluated by the parameters coming from the record force–distance [1], from the elastic recovery test [2], the creep test [3] or the stress relaxation test [4]. Stress relaxation is the test where the tablet is pressed up to a certain compacting pressure and after reaching this pressure the punches are stopped for some time period. At this moment the decrease in compacting pressure is measured. David and Augsburger [5] analyzed stress relaxation using the single Maxwell model and they tried to quantify the range of plastic deformation for several fillers. Rees and Rue [6] applied stress relaxation with a 30 s dwell time and they found that only one Maxwell model was not suitable for the description of the pressure drop during the dwell time. The Maxwell model is a combination of Hooke's and Newton's bodies in series. The authors evaluated the plasticity of five fillers only on the basis of the pressure differences at the beginning and at the end of the dwell time. Ebba et al. [7] used the stress relaxation test with a 60 s dwell time to assess the influence of lubricants on the compatibility of some fillers. These authors characterized the relation of decreasing pressure in time by three and four exponential equations, where each term corresponded to one Maxwell model. The value of Newton's body was calculated for every Maxwell model. Three exponential equations were used and described also by Manas and Salil [8].

(1) $$CP\text{(}t\text{)}=A_{1}e^{-\frac{t}{T_{1}}}+A_{2}e^{-\frac{t}{T_{2}}}+A_{3}e^{-\frac{t}{T_{3}}}+A_{0}$$

Eq. (1) corresponds to three Maxwell models combined with a Hook body set in parallel and having common deformation. Each term of the exponential equation represents one of the three processes describing the pressure decrease in a tablet. The individual values A_i_ (Pa) mean the extent of the pressure drop in a material during stress relaxation at a certain process. A_0_ is the pressure remaining in the material after the dwell time, t (s) is real time and T_i_ (s) is the relaxation constant defined as the time required for decreasing pressure to relax to a stress of a magnitude of 1/e. The values of A_i_ simultaneously express the parameters of elasticity.

The modulus of plasticity P_i_ (Pas) is calculated using Eq. (2):

(2) $$P_{i}=A_{i}*T_{i}\text{.}$$

Eq. (1) and its elastic and plastic parameters have been used up to now only for the assessment of viscoelastic properties of various potato cultivars [9]. In the tablet area, a similar interpretation of the moduli of the three exponential equations has not been carried out yet. The authors have attempted only to generally explain what happens inside a tablet at stress relaxation. Cole et al. [10] shows that during the dwell time plastic deformation proceeds, whereas a shift of particles to free spaces and a more intensive bond formation occur. Maarschalk et al. [11] emphasize the fact that the modulus values of stress relaxation depend on the amount of energy stored in a tablet during compaction and on bond formation. Lum and Duncan-Hewitt [12] classify three processes of behavior of standard solid materials at stress relaxation. The first process expresses the initial elastic response to the load used or eliminated, the second one is the retarded elastic response, and the third one is then the permanent deformation during the plastic flow. This method characterizes the viscoelastic behavior of tablets during compaction.

## 2 Materials

Model pharmaceutical fillers for direct compaction were calcium hydrogen phosphate dihydrate Emcompress (DHCP) manufactured by the firm JRS Group (lot 1048, United Kingdom), lactose monohydrate 200 (LMH) manufactured by the firm Meggle AG (lot L0639A4950, Germany), mannitol Pearlitol 200 SD (MAN) manufactured by the firm Roquette (lot E172P, France), microcrystalline cellulose Avicel PH 102 (MCC) manufactured by the firm FMC Corporation (lot 6610260938, Belgium), cellulose powder Vitacel A 300 (CEL) manufactured by the firm J. Rettenmaier (lot 0708050429, Germany), and hydroxypropyl methylcellulose K4 (HPMC) manufactured by the firm Dow Chemical (lot UH20012N11, Belgium). All materials comply with the *European Pharmacopoeia* and were used without any adjustment.

## 3 Methods

### 3.1 Evaluation of particles

Volumes and densities of materials used were measured on a machine SVM 102 (Erweka, Heusenstamm, Germany) to calculate Carr's index [13] and Hausner's ratio [14]. The particle size was determined by mesh analysis on a shaker (Mechanical Engineering, Brno, Czech Republic). Each of the materials was shaken for 5 min through seven sieves.

### 3.2 Preparation of tablets

Tablets of the diameter of 13 mm and weight of 500 mg with the precision of +/−0.001 mg were compacted in the compaction punches (Adamus HT, Machine Factory Group, Szczecin, Poland) in a device for testing the strength of materials in tension and pressure T1-FRO 50 (Zwick GmbH, Ulm, Germany). Tablets were compacted with the following adjustment of the machine: distance of jaws, 117 mm, rate of cycle, 2 mm/s, pre-load, 2 N, the compaction pressures of 1.9 MPa, 3.8 MPa, 7.5 MPa, 150 MPa, 22.6 MPa, 30.1 MPa, 37.7 MPa, 56.5 MPa, 75.3 MPa, 942 MPa, and 113.0 MPa. In order to determine stress relaxation, tablets were compacted with a pause of 180 s. During the pause, the punch was stopped at the position where it achieved the maximal force, and the decrease in force of the upper punch in time was recorded. In each compaction force, 6 tablets were evaluated.

### 3.3 Stress relaxation test

Two methods were employed for the calculation of the stress relaxation test parameters.

The first method used the ratio CS_max_/CS_0_ [15] as the viscoelastic parameter. CS_max_ is the maximum compaction pressure at the beginning of the dwell time and CS_0_ is the pressure at the end of the dwell time. The tablets were compressed at 75.3 MPa.

The second method used three exponential equations for calculation of parameters of pressure decrease in a tablet at a 180 s dwell time (1).

(1) $$CP\text{(}t\text{)}=A_{1}e^{-\frac{t}{T_{1}}}+A_{2}e^{-\frac{t}{T_{2}}}+A_{3}e^{-\frac{t}{T_{3}}}+A_{0}$$

The parameters of the above-mentioned equation were calculated by means of the OriginPro 7.5 software using the ExpDec3 function. CP (MPa) is the compacting pressure at time t (s), A_1–3_ (MPa) is the pressure decrease at a given process and it expresses the elastic parameter as well, T_1–3_ is the relaxation constant indicating the rate and slope of the process, and A_0_ (MPa) is the pressure remaining in a tablet after the dwell time.

The modulus of plasticity P (MPas) was expressed for every compaction pressure as follows (2):

(2) $$P_{i}=A_{i}*T_{i}\text{.}$$

The higher the value of the relaxation constant, the higher then the modulus of plasticity. The value of the pressure inside the tablet at a high T_i_ reduces slowly and is fully utilized for bond formation.

As the moduli of elasticity and plasticity characterize only the status at one compaction pressure, the parameter of total elasticity and plasticity was introduced.

The total elasticity A_Ti_ (MPa) equals to the area under the plot A/CP vs. CP. The total plasticity P_Ti_ (MPas) then corresponds to the area under the curve P/CP vs. CP.

### 3.4 Statistical analysis

The results of the stress relaxation tests were evaluated by a one-way analysis of variance ANOVA using the Origin program version 7.5. Results were quoted as significant when p < 0.05.

## 4 Results and discussion

### 4.1 Particle evaluation

**Fig. 1:**
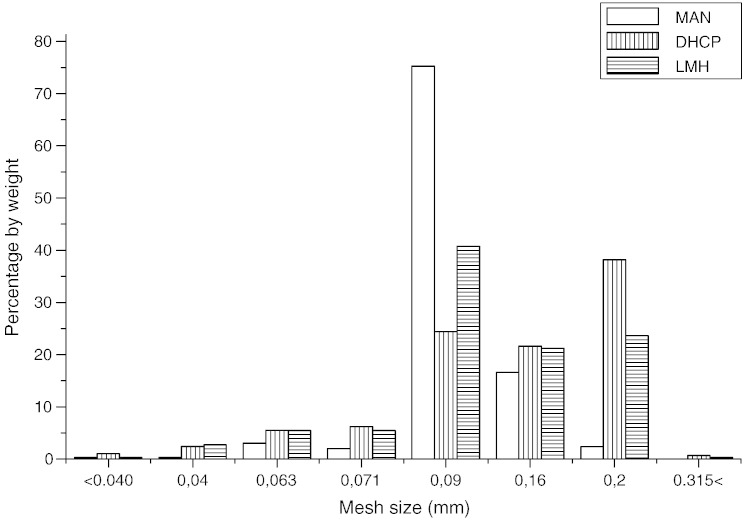
The particle size distribution of MAN, DHCP and LMH.

**Fig. 2:**
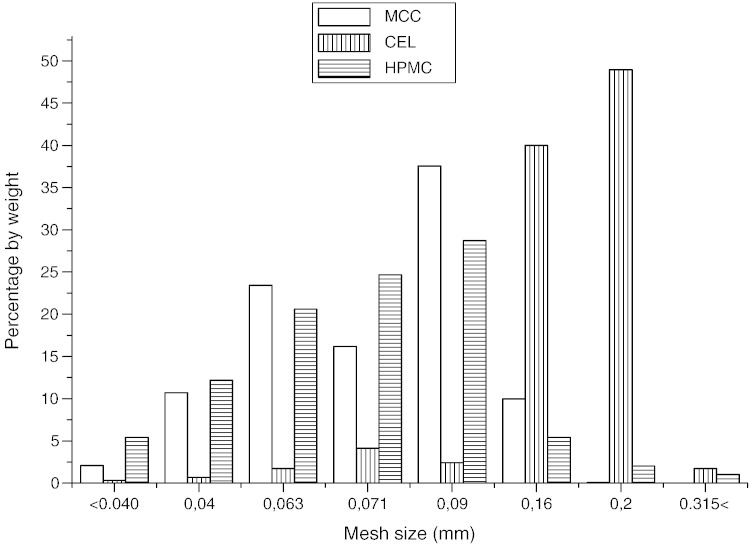
The particle size distribution of MCC, CEL and HPMC.

Particle size of the excipients studied was evaluated by mesh analysis (Table 1, Figs. 1 and 2). CEL had the largest particle size — 181 μm. On the contrary, the cellulose derivates showed substantially smaller particles — MCC 80 μm and HPMC 74 μm. From crystalline excipients, DHCP had the largest particles — 153 μm, LMH had slightly smaller particles — 120 mm and finally MAN — 108 μm.

The Hausner ratio characterizes the flowability of the materials compressed. Materials having the ratio below 1.25 suit for the preparation of tablets [16]. The remaining fillers studied showed good flowability. The same conclusion can be made from the values of Carr's index.

### 4.2 Stress relaxation test

The values of the ratio CS_max_/CS_0_ express the behavior of fillers during compressing. If the value of this ratio is higher than 1.3, the fillers are ductile. Fragmenting materials have this ratio about 1.00 [15]. The results show that MCC (1.30), CP (1.31), HPMC (1.36) and MAN (1.38) are ductile. On the other hand, LMH (1.12) and DHP (1.10) are fragmenting materials. Narayan and Hancock came to the same conclusion.

Using the ratio CS_max_/CS_0_ for the evaluation of viscoelasticity, only one parameter is obtained. This parameter assesses only the stage after the compaction and is not able to describe the entire compacting process. The process of decrease in compacting pressure according to this equation is classified into three processes. The individual processes are evaluated by means of elastic and plastic parameters. The model (Eq. (1)) expresses the elasticity parameter A as an extension of a spring and simultaneous decrease in its energy. The plasticity parameter P implies a compression of a dashpot. The rate of the dashpot compression is the response to the energy coming from the extension of the spring. The more the dashpot is compressed, the larger the number of bonds between compressed particles is formed.

The results of the elasticity parameters A_T1–3_ and plasticity parameters P_T1–3_ are presented in Tables 2 and 3.

Viscoelastic behavior of model fillers during compression is influenced by their ability to form various types of bonds. DHCP is able to create van der Waals bonds [17]. Their presence was followed by means of AFM, e.g. in literature [18]. MAN a LAC could form solid bridges [19]. Solid bridges are proposed to develop by melting, diffusion of atoms between surfaces or recrystallization of soluble materials in the compacts. Presence of moisture is also important in the formulation of solid bridges [20]. Hydrogen bonds occur at compression of celluloses and their derivatives [21]. Hydrogen bonds are formed between molecules containing electronegative atoms (O, N, F, Cl) and hydrogen atoms [22]. E.g. literature [23], deals with hydrogen bonds in celluloses and follows their presence by FTIR.

**Fig. 3:**
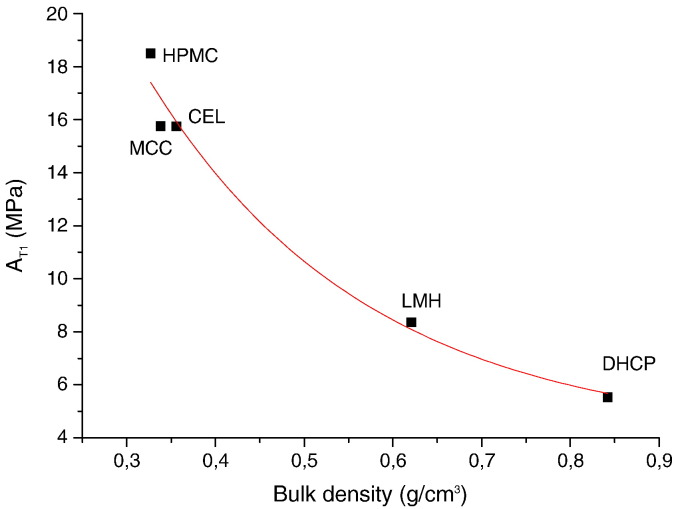
The relationship between parameter A_T1_ and bulk density.

In the first process, the tablet particles react to punch stop by instantaneous elastic response. During the dwell time the particles elastically deformed have a tendency to recover their original shape and volume. They expand and fill free spaces in the tablet [12]. This first process takes only about 5 s and acting elastic forces are rapidly minimized. The elasticity parameter A_T1_ reaches higher values compared to A_T2_ and A_T3_. We assume that the majority of elastic forces are consumed in the case of low-density and low-friction materials [12]. The decrease in bulk density led to a higher value of the parameter A_T1_ (Fig. 3) except MAN. The behavior of MAN differed due to its melting and recrystallization during compaction. The relaxation constant T_1_ was used to calculate the plasticity parameter P_T1_. There was no significant difference in T_1_ between individual excipients (p = 0.102), except DHCP (p = 0.025). The values of PT_1_ were very low and ranged between 1.860 MPas–5.116 MPas and had no effect on bond formation.

HPMC reached the highest parameter of elasticity — 18.503 MPa. There was a statistically significant difference in the elasticity parameter A_T1_ (p = 0.031) between HPMC and other celluloses. On the other hand, there was no significant difference in A_T1_ between MCC and CEL. The same can be said about the plastic parameter P_T1_ where a statistically significant difference (p = 0.015) for HPMC in contrast to the remaining two polymers was found. HPMC has a lower density than MCC and CEL and this fact offers more space for elastic expansion of particles. Elastic energy stored in a low-density tablet is better consumed than in a high-density one. The lowest density of HPMC results in the highest P_T1_ — 5.030 MPas.

The crystalline compounds show the differences in elasticity moduli (p = 0.012). The excipient DHCP had the lowest parameter A_T1_. It may be caused by its high density — the particles have no space for shape recovering and a high friction prevents particles from any moving. The elastic energy which was consumed in the first process by the other materials was stored in DHCP to the end of compaction. Elastic energy consumed in the case of other substances in the first process remains stored in DHCP even after completion of compaction.

LMH behaved like DHCP. Nevertheless, some portion of the amorphous part enables better particles rearrangement and that is the reason for a higher A_T1_.

MAN reached very high values of A_T1_, so that it seems to be a plastic material. However, MAN melts at compaction pressures higher than 7.5 MPa. Melting of particle consumed the main portion of elastic energy, which resulted in high values of A_T1_.

MAN had the highest P_T1_ value. DHCP had a higher value of P_T1_ than LMH but this difference is not statistically significant (p = 0.809).

**Fig. 4:**
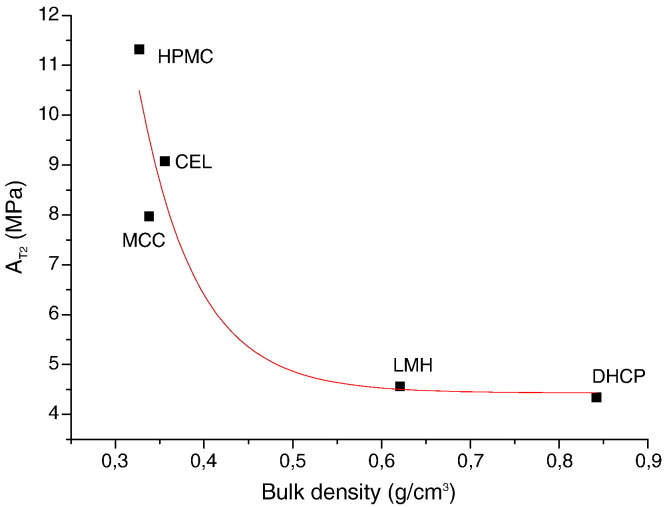
The relationship between parameter A_T2_ and bulk density.

Lum and Duncan-Hewitt [12] described the second process as a retarded elastic response. Particles try to recover its original volume but with a lower rate. The free space in tablets is filled and the particle–particle interactions occur more intensively than in the first process. This second process runs approximately for 25 s. The elastic parameters A_T2_ are lower than A_T1_. The value of the parameter A_1_ increased exponentially with a decrease of tapped density. However, MAN is an exception (Fig. 4). No significant difference was found (p = 0.102) for relaxation constants T_2_, except DHCP (p = 0.011).

Low density and existence of small particles which are able to rearrange are the reason for high elastic parameter A_T2_ (11.319 MPa) of HPMC. A significant difference was between CEL (A_T2_ = 9.074 MPa) and MCC (A_T2_ = 7.973 MPa). Explanation of this phenomenon might be that larger and irregular particles of CEL are able to form mechanical interlocking [17]. The high capability of HPMC to form hydrogen bonds [21] leads to a high P_T2_ value (43.527 MPas). The difference in the P_T2_ (p = 0.001) values of MCC (25.014 MPas) and CEL (33.342 MPas) is surprising. CEL forms probably more hydrogen bonds than MCC in this phase.

Situation of DHCP and LMH is similar as in the first process. The drop of elastic forces is low because of high density and friction of particles.

Difference in elastic moduli A_T2_ is insignificant (p = 0.072). On the contrary, MAN has a high A_T2_ thanks to low friction in the melt.

MAN makes up bonds called solid bridges [19]. It results in a high value of P_T2_ (45.859 MPas). A big difference exists between LMH and DHCP. P_T2_ of DHCP is 27.111 MPas, which is a much higher value than that of LMH (16.194 MPas). DHCP forms weak van der Waals bonds [17]. DHCP has a significantly higher value of relaxation constant T_2_, which gives longer time for bond formation.

The third process is described as permanent plastic deformation [12]. The particles have no possibility of volume growth or to move. There is already a close contact with other particles. The decrease in elastic forces is lowest in this third process. This process takes about 150 s. The exception to this rule is again MAN. The first and second processes mean the highest reduction of elastic energy, which could damage the tablet structure after its ejection from the die. Stabilization (bond formation) takes place in this third process.

No significant differences (p = 0.112) were found for T_3_ values of the excipients studied.

The magnitude of elastic moduli A_T3_ does not differ from A_T2_ even though the third process is six times longer than the second one. Particles do not practically move and expand. The order of the elastic moduli A_T3_ and A_T2_ for polymers is identical. HPMC in large numbers forms hydrogen bonds between hydroxypropoxy and methoxy groups. HPMC has the highest P_T3_ (576.813 MPas). CEL makes up hydrogen bonds as well, but its value of P_T3_ (557.967 MPas) is statistically less significant (p = 0.003). MCC has a low value of P_T3_ (411.648 MPas). This filler forms very hard tablets when pressed even at low compaction pressures and expending bonding potential before the dwell time.

MAN has the highest values of A_T3_ and P_T3_ in the group of crystalline compounds. It is brought about by the existence of solid bridge bonds. Much more interesting and surprising is the value of A_T3_ of DHCP (6.778 MPa), which is much higher than that of LMH and comparable with A_T3_ of MCC (6.884 MPA, p = 0.057). The values of P_T3_ DHCP are statistically the same as those for MCC (p = 0.976). A high P_T3_ indicates extensive formation of van der Waals bonds. These weak bonds are formed on the interparticle surfaces and they are interrupted after compression of tablets due to the elastic forces still remaining in the tablet. It is the reason why the DHCP tablets have low hardness and tend to cap even though P_T3_ is high. Remaining elastic forces damage the tablet structure after ejection from the die.

LMH has a very low A_T3_, which is in accordance with our expectation. P_T3_ is very low as well, even though the amorphous part of LMH is described as plastic [17]; a low number of bonds are formed.

**Fig. 5:**
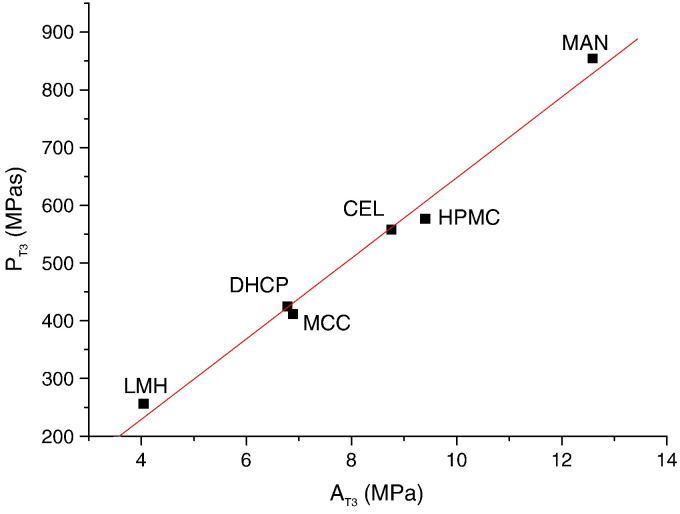
The relationship between parameter A_T3_ and P_T3_.

A relation was found between elastic and plastic parameters of the individual processes, whereas no linear dependence was found between the first and second process. The coefficient of correlation of the function A_T1_ and P_T1_ had the value 0.922; A_T2_ and P_T2_ 0.887. The pressure A_T1–2_ was consumed for filling of the free space and it was very different for individual excipients. A linear relation between A_T3_ and P_T3_ exists in the third process (Fig. 5). This relation can be expressed by the equation

(3) $$P_{T3}=-49.918+69.789A_{T3}$$

$$\left(r=0.9942\right)\text{.}$$

This finding means that the plastic parameter increased linearly with an increasing value of the elastic parameter. The excipients studied in the third process differ only in the magnitude of the elastic parameter. The use of this energy depends on the type and number of bonds formed at certain excipients.

## 5 Conclusions

Three simultaneously running processes during the stress relaxation can be mathematically separated by the three exponential Eq. (1). Each term of equation gives us more details about elastic and plastic stages under constant volume (instant elastic deformation, retarded elastic deformation and permanent plastic deformation) [12]. The elastic parameters point out that an important factor is bulk density. In the early stage of the stress relaxation, testing the release of elastic forces is facilitated by low bulk density (high value of elastic parameters) and these forces will not contribute to capping after tablet ejection.

The plastic parameters illustrate particles tendency to form bonds. In the first and second processes particle–particle interactions start occurring. The most bonds are formed during the third process where particles are in closest contact.

Various values of elastic and plastic parameters show clearly differences between used materials and such an approach explaining and quantifying the structure influence has not been realized yet.

## Figures and Tables

**Table 1:** Basic characteristics of excipients.

	Particle diameter (mm)		CI (%)		HP		Bulk density (g/cm^3^)	
	x	s	x	s	x	s	x	s
MCC	0.080	0.003	26.300	1.178	1.353	0.021	0.338	0.008
CEL	0.181	0.004	19.567	0.834	1.240	0.014	0.356	0.012
HPMC	0.074	0.001	28.600	0.920	1.393	0.019	0.327	0.010
MAN	0.108	0.002	13.933	0.047	1.160	0.000	0.505	0.015
LMH	0.120	0.002	15.000	1.273	1.170	0.014	0.621	0.005
DHCP	0.153	0.005	12.000	0.047	1.211	1.211	0.842	0.007

**Table 2:** The values of total elasticity A_T1–3_.

	A_T1_ (MPa)		A_T2_ (MPa)		A_T3_ (MPa)	
	x	s	x	s	x	s
MCC	15.758	0.213	7.973	0.101	6.884	0.088
CEL	15.754	0.207	9.075	0.112	8.756	0.096
HPMC	18.503	0.219	11.320	0.133	9.397	0.119
MAN	18.223	0.262	11.048	0.125	12.587	0.129
LMH	8.365	0.180	4.563	0.055	4.043	0.057
DHCP	5.532	0.097	4.340	0.063	6.778	0.091

**Table 3:** The values of total plasticity P_T1–3_.

	P_T1_ (MPas)		P_T2_ (MPas)		P_T3_ (MPas)	
	x	s	x	s	x	s
MCC	3.2493	0.1348	25.0141	1.3365	411.6479	5.4565
CEL	3.9833	0.1589	33.3424	2.0155	557.9670	9.1853
HPMC	5.0296	0.2412	43.5275	2.1255	576.8125	8.9522
MAN	5.1160	0.3654	45.8595	2.3564	854.1947	14.5681
LMH	1.8603	0.0215	16.1939	0.9512	255.9144	4.1534
DHCP	2.1529	0.0870	27.1113	1.6584	424.8550	15.8260
